# Identification and Differential Abundance of Mitochondrial Genome Encoding Small RNAs (mitosRNA) in Breast Muscles of Modern Broilers and Unselected Chicken Breed

**DOI:** 10.3389/fphys.2017.00816

**Published:** 2017-10-20

**Authors:** Walter G. Bottje, Bhuwan Khatri, Stephanie A. Shouse, Dongwon Seo, Barbara Mallmann, Sara K. Orlowski, Jeonghoon Pan, Seongbae Kong, Casey M. Owens, Nicholas B. Anthony, Jae K. Kim, Byungwhi C. Kong

**Affiliations:** ^1^Department of Poultry Science, Center of Excellence for Poultry Science, University of Arkansas, Fayetteville, AR, United States; ^2^School of Human Environmental Sciences, University of Arkansas, Fayetteville, AR, United States

**Keywords:** mitosRNA, mitochondria, chicken breast muscle, small RNA sequencing, mitochondrial contents

## Abstract

**Background:** Although small non-coding RNAs are mostly encoded by the nuclear genome, thousands of small non-coding RNAs encoded by the mitochondrial genome, termed as mitosRNAs were recently reported in human, mouse and trout. In this study, we first identified chicken mitosRNAs in breast muscle using small RNA sequencing method and the differential abundance was analyzed between modern pedigree male (PeM) broilers (characterized by rapid growth and large muscle mass) and the foundational Barred Plymouth Rock (BPR) chickens (characterized by slow growth and small muscle mass).

**Methods:** Small RNA sequencing was performed with total RNAs extracted from breast muscles of PeM and BPR (*n* = 6 per group) using the 1 × 50 bp single end read method of Illumina sequencing. Raw reads were processed by quality assessment, adapter trimming, and alignment to the chicken mitochondrial genome (GenBank Accession: X52392.1) using the NGen program. Further statistical analyses were performed using the JMP Genomics 8. Differentially expressed (DE) mitosRNAs between PeM and BPR were confirmed by quantitative PCR.

**Results:** Totals of 183,416 unique small RNA sequences were identified as potential chicken mitosRNAs. After stringent filtering processes, 117 mitosRNAs showing >100 raw read counts were abundantly produced from all 37 mitochondrial genes (except D-loop region) and the length of mitosRNAs ranged from 22 to 46 nucleotides. Of those, abundance of 44 mitosRNAs were significantly altered in breast muscles of PeM compared to those of BPR: all mitosRNAs were higher in PeM breast except those produced from 16S-rRNA gene. Possibly, the higher mitosRNAs abundance in PeM breast may be due to a higher mitochondrial content compared to BPR. Our data demonstrate that in addition to 37 known mitochondrial genes, the mitochondrial genome also encodes abundant mitosRNAs, that may play an important regulatory role in muscle growth via mitochondrial gene expression control.

## Introduction

Production efficiency in animal agriculture is critically important in order to meet the increasing needs of high quality meat protein of a growing global human population. Modern broiler chickens have been highly selected for rapid growth and feed efficiency compared to unselected (progenitor or heritage) chicken breeds. Consequently, the understanding of key regulatory mechanisms for rapid muscle growth and enhanced feed efficiency is imperative with regard to more efficient, and therefore sustainable, food animal production (Niemann et al., 2011).

Global gene expression studies using cDNA microarray assay (Kong et al., 2011; Bottje et al., 2012; Bottje and Kong, 2013), RNA sequencing (Bottje et al., 2017), and shotgun proteomics (Kong et al., 2016) have been carried out on breast muscle obtained from pedigree males (PeM) exhibiting either a high or low feed efficiency (FE) phenotype. Additionally, global transcriptomics has been conducted on breast obtained from PeM broilers (characterized by rapid growth, large muscle mass and higher feed efficiency) compared with the foundational Barred Plymouth Rock (BPR) chicken breed (exhibiting slow growth, small muscle mass and lower feed efficiency) (Kong et al., 2017). The BPR chicken exhibits a characteristic pattern of alternating white and black bars of feather pigmentation and was developed in the United States during the mid-nineteenth century (Dorshorst and Ashwell, 2009). The BPR breed was developed for dual purposes of both meat and egg production and has a much slower growth rate compared to commercial broilers (Lopez et al., 2007). Several global expression studies showed that production efficiency may be associated with various cellular mechanisms including mitochondrial oxidative stress, myogenic growth and differentiation, inflammatory response, protein degradation, stress responses, growth hormone signaling, cell cycle, apoptosis, and fatty acid transportation. Particularly, the association between production efficiency and mitochondrial functions on energy expenditure and oxidative phosphorylation system (OXPHOS) in chicken breast muscle has been well-documented (Bottje et al., 2002, 2006, 2009; Bottje and Carstens, 2009; Bottje and Kong, 2013).

A major function of mitochondria is in producing energy (ATP) through OXPHOS in most eukaryotic cells (Taylor and Turnbull, 2007). Mitochondria contain their own mitochondrial DNA (mtDNA) that exhibits characteristics that differ from nuclear DNA, including divergent genetic codes, transmission by maternal inheritance of mitochondrial type, higher rates of mutation due to proximity of mtDNA to the electron transport chain, polyploidy status, and a more compact organization (Fernandez-Silva et al., 2003). Similar to mammalian mitochondrial genome, chicken mtDNA harbors one control region (D-loop) and contains 37 genes encoding 2 rRNA subunits (12S and 16S rRNAs), 22 transfer RNAs (tRNAs) and 13 proteins that are all involved in OXPHOS (Zhao et al., 2016).

In addition to mitochondrial RNAs, mtDNA encoding small RNAs (mitosRNAs) were recently discovered in human, mouse and rainbow trout (Ro et al., 2013; Ma et al., 2016). These novel mitosRNAs are hypothesized to regulate mitochondrial gene expression and mRNA stability (Ro et al., 2013). While mitosRNAs have been detected in mammalian cells, these have not been characterized in avian species to our knowledge. Therefore, the present study was conducted to identify mitosRNAs in avian breast muscle and to determine if differences in abundance may occur in birds exhibiting large differences in growth and muscle development in a line of birds highly selected for growth and development (PeM broilers) and a heritage breed, BPR chickens.

## Materials and methods

### Ethics statement

The present study was performed in accordance with the recommended guidelines for the care and use of laboratory animals of the National Institutes of Health. All procedures for animal care were followed by animal use protocol that were reviewed and approved by the University of Arkansas Institutional Animal Care and Use Committee (IACUC Protocol #14012).

### Breast muscle tissue and RNA extraction

Pedigree male broilers within a single genetic line and RNA extraction were described previously (Bottje et al., 2002; Kong et al., 2017). Briefly, PeM and BPR chickens (6–8 weeks old, *n* = 6 per breed) were humanely killed by cervical dislocation. Breast muscle tissue was rapidly excised and immediately flash frozen in liquid nitrogen. Total RNA was isolated from the tissue using TRIzol reagent (Invitrogen Life Technologies, Thermo-Fisher Scientific, Carlsbad, CA) according to the manufacturer's instructions. After initial extraction, the RNA samples were treated with DNase I (New England Biolabs Inc., Ipswich, MA) and extracted a second time with TRIzol reagent. Assessing RNA quality using an Agilent 2200 TapeStation instrument (Santa Clara, CA) revealed that all RNA samples showed >8.0 values of RNA integrity number (RIN). These RNA samples were then used for small RNA sequencing (RNAseq) analysis outlined below.

### RNAseq and data analysis

Small RNAseq library preparation for individual samples with barcoding and Illumina sequencing was carried out at the Research Technology Support Facility (Michigan State University, East Lansing, MI). The Illumina HiSeq system at this facility used a 1 × 50 bp single end read technology for sequencing the RNA samples. The RNA sequence FASTQ files that were obtained were mapped to the chicken mitochondrial genome (GenBank Accession: X52392.1) using the NGen program in Lasergene software package (DNAStar, Madison, WI). The total mapped counts were transformed to log_2_ values based on the number of reads per million (RPM) in order to stabilize the variance. The resulting normalized values were then subjected to further statistical analyses using the JMP Genomics 8 (SAS Institute Inc., Cary, NC). The small RNAs aligned to mitochondrial genome showing >100 average read counts derived by six individual samples, adjusted *p*-value (false discovery rate; FDR) <0.05 after *t*-test and log_2_ fold change >0.5 were considered as differentially expressed (DE) between PeM and BPR. The *p*-value correction (FDR calculation) was performed by multiple tests of Benjamini and Hochberg method (1995) using JMP Genomics 8.

### Nomenclature of identified mitosRNAs

The mitosRNA sequences were identified based on previous reports (Ro et al., 2013; Ma et al., 2016), and their naming was simplified somewhat to contain five parts: [species]-mitosRNA-[strand]-[location]-[variants/fragments]. As an example, gga-mitosRNA-H-2344-1 refers to chicken mitochondrial small RNA derived from the 2344 locus on the heavy strand of the first fragment, whereas gga-mitosRNA-L-16707 refers to chicken mitochondrial small RNA derived from the 16707 locus on the light strand. If multiple fragments determined to have been derived from the same gene locus, the fragments were sequentially numbered.

### Quantitative PCR of mitosRNA

Quantitative PCR (qPCR) was conducted following the method reported previously (Ro et al., 2013; Ma et al., 2016) with modifications. Briefly, 60 μg of total RNA obtained from 6 muscle samples from PeM and BPR breast muscle was used for general validation of the RNAseq results and for specific confirmation of the most abundant mitosRNAs for each mitochondrial gene region. Small RNAs were purified from total RNA samples using a miRNA isolation kit (mirVana, Thermo Fisher Scientific, Waltham, MA), polyadenylated with a *E. coli* Poly-A Polymerase (Thermo Fisher Scientific) and reverse transcribed to cDNA using an adapter primer containing poly T residues at 3′-end (Table 1) and SuperScript III reverse transcriptase (ThermoFisher Scientific) following the manufacturer's instructions. The resulting cDNA samples were diluted (1:10) and a portion (2 μL) was used for qPCR determination (total volume of 25 μL) with an ABI prism 7500HT system (ThermoFisher Scientific) with PowerUp™ SYBR® Green Master Mix (ThermoFisher Scientific). Specific oligonucleotide primers for individual mitosRNA and the universal reverse primer are shown in Table 1. The conditions of real-time qPCR amplification were as follows: 1 cycle at 95°C (10 min), 40 cycles at 95°C (15 s each), followed by 60°C for 1 min. The chicken small 5S rRNA gene was used as the internal control. All qPCR reactions were conducted in duplicate and the values of average cycle threshold (Ct) determined for each sample (*n* = 6 for each PeM and BPR). The 2^−ΔΔCt^ values were calculated using the average ΔCt (PeM) − average ΔCt (BPR) indicating that the BPR group values were set to 1 and used for relative quantification by linear fold-change. The statistical significance (*p* < 0.05) between PeM and BPR values were determined by *t*-test.

**Table 1:** Primers used for qPCR.

**Primer name**	**Primer sequence**
gga_mitosRNA_H_1781_F	ACATGTATCCGCCTGAGAACT
gga_mitosRNA_H_2346_6_F	TCTAGCCCGACAAACTCGTA
gga_mitosRNA_H_4040_F	ACATGACCCTGCCCACCCTAA
gga_mitosRNA_H_4957_F	ATGCCTCTGACATACCAGCAT
gga_mitosRNA_H_8083_F	AAGTACTCCAACCCGAATTA
gga_mitosRNA_H_8272_F	ACCAATTACATAGACCTGTC
gga_mitosRNA_H_8487_F	AGATGCCCAAGAAGTTGAAC
gga_mitosRNA_H_9163_F	TCACTCTAACAAACAACCCT
gga_mitosRNA_H_10617_F	TCGGATTTGAAGCAGCAGCCT
gga_mitosRNA_H_11350_F	ACCCCATCATTCGCCCTTGT
gga_mitosRNA_H_11664_F	TCAACTCCCCTCTTAGTACT
gga_mitosRNA_H_15821_F	AACGAACAATAACCTTCCGA
5S_rRNA-F1	AAGCCTACAGCACCCGGTAT
RTQ-UNI_R	CGAATTCTAGAGCTCGAGGCAGG
RT-Primer with adapter	CGAATTCTAGAGCTCGAGGCAGGCGACATGG CTGGCTAGTTAAGCTTGGTACCGAGCTCG GATCCACTAGTCCT TTTTTTTTTTTTTTTTTTTTTTTTVN
ND1_F	ACCCTAGCCATCATCCTGTT
ND1_R	TCCTGAGACTAGCTCTGACT
ND2_F	AGCATAACCAACGCCTGATC
ND2_R	GATGTTAGGAGGAGGAGTGT
COX1_F	TCCTTCTCCTACTAGCCTCA
COX1_R	AGGAGTAGTAGGATGGCAGT
COX2_F	AGGCTTTCAAGACGCCTCAT
COX2_R	GTGAGATCAGGTTCGTCGAT
ATP8_F	ATGCCCCAATTAAACCCTTTCCCA
ATP8_R	TTAGGTTCATGGTCAGGTTCA
ATP6_F	AATTCTCAAGCCCCTGCCTA
ATP6_R	AGGAGGCCTAGGAGGTTAAT
COX3_F	TAGTTGACCCAAGCCCATGA
COX3_R	GTAGGCCCTTTTGGACAGTT
ND3_F	TCTACTAAGCGCTGCACTAA
ND3_R	AGGGCGATTTCTAGGTCGAA
ND4L_F	TCCCCTACACTTCAGCTTCT
ND4L_R	TTCGCATGCTGAGAAGGCTA
ND4_F	TCGATCAGCCTACACTGACT
ND4_R	TGGGATTAGGGTTGCTTCGA
ND5_F	AGCCTCAATGGAGAACAAGACA
ND5_R	TGTGGCAAGTAGTGTAAGTGA
CYTB_F	TGCCTCATGACCCAAATCCT
CYTB_R	AGTGTGAGGAGGAGGATTACT
ND6_F	AGACAACCCACGCACAAGCT
ND6_R	CTAGGTTTTGTCTTGGTGGT
GAPDH_F	ACAGCAACCGTGTTGTGGAC
GAPDH_R	CAACAAAGGGTCCTGCTTCC

*The first column indicates primer names and the second column shows sequences for the forward and reverse primers*.

### Determination of mitochondrial contents using qPCR

Total DNAs of muscle samples were purified using Zymo Tissue and Insect DNA mini Prep kit (Zymo Research, Irvine, CA). Ten nanogram of total DNA were subjected to qPCR reaction with primers for mitochondrial genes that included; (a) NADH-ubiquinone oxidoreductase chain (ND): ND1, ND2, ND3, ND4, ND5, ND6, (b) cytochrome oxidase (COX): COX1, COX2, COX3, (c) cytochrome b (CYTB), and (d) ATPase (ATP) 6, and ATP8, as well as nuclear encoded genes (glyceraldehyde-3-phosphate dehydrogenase; GAPDH) using an ABI prism 7500HT system (ThermoFisher Scientific) with PowerUp™ SYBR® Green Master Mix (ThermoFisher Scientific). The specific oligonucleotide primers are listed in Table 1 and quantifying methods described above.

## Results and discussion

### Identification of breast muscle MitosRNAs in chicken

Twelve small RNAseq libraries were constructed using RNA isolated from breast muscles obtained from PeM and BPR chickens (*n* = 6 per group) and sequenced with the 1 × 50 bp single end read method. A total of ~41 million 50 bp sequence reads were produced with an average of ~3.3 million reads per sample (data not shown). After quality assessment, adapter trimming of raw reads, and alignment to chicken mitochondrial genome (GenBank Accession: X52392.1) using the NGen program, ~1.4 million reads with an average of ~120,000 reads per sample (~350 × average coverage) were aligned to the entire sequence of the mitochondrial genome (data not shown). About 97% of reads used in alignment were in the sense orientation of the mitochondrial genome (data not shown). Totally 183,416 unique small RNA sequences were identified as potential chicken mitochondrial genome encoding small RNAs (mitosRNAs) (data not shown). After stringent filtering, 117 mitosRNAs showing >100 raw read counts were abundantly produced from all 37 mitochondrial genes (except D-loop region), all of them were in sense orientation, and the length of mitosRNAs ranged from 22 to 46 nucleotides (Supplementary Table 1).

**Figure 1:**
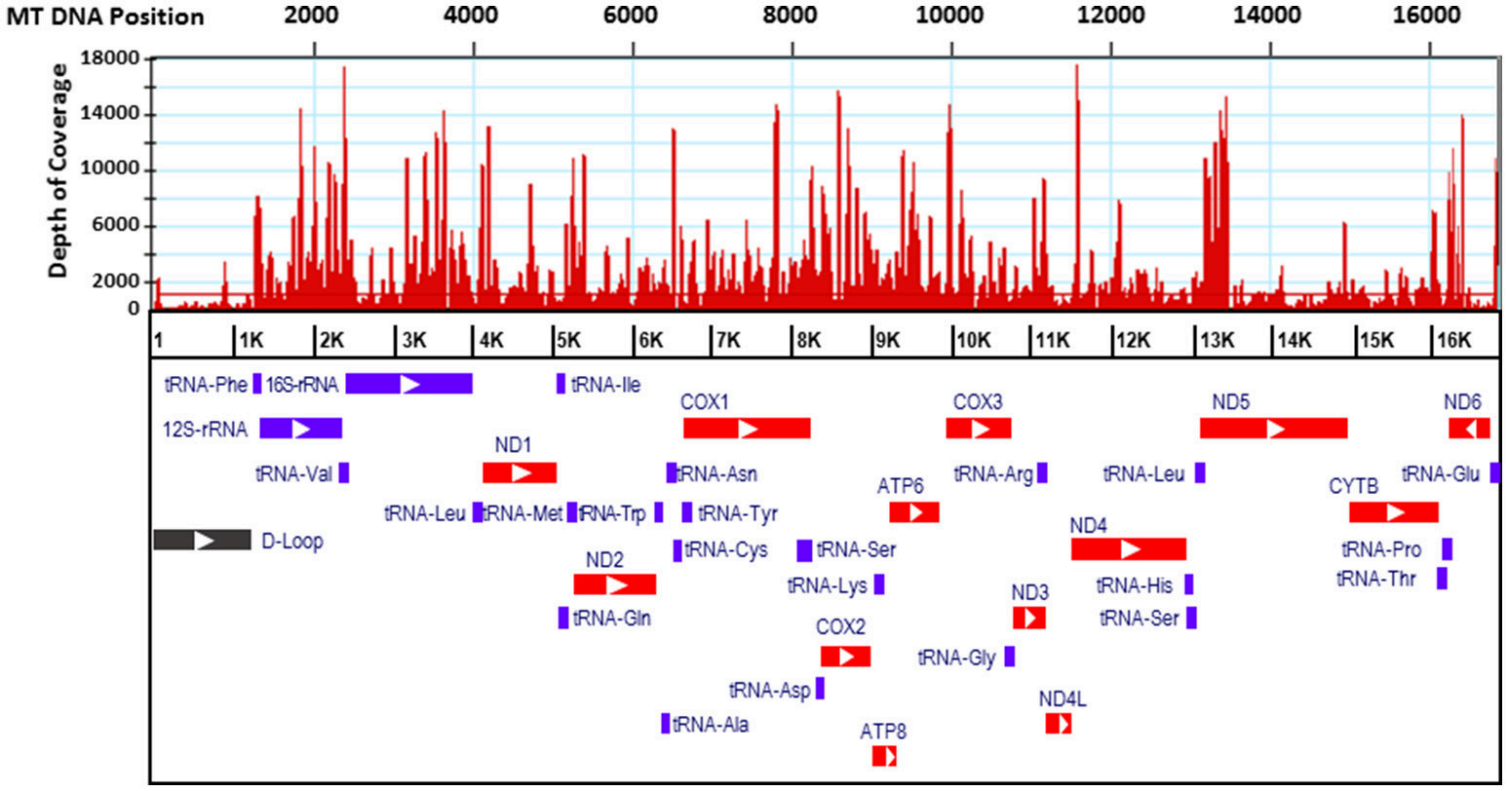
Distribution of mitosRNA in the chicken mtDNA regions. Red histograms indicate abundance level of mitosRNAs. Relative location of genes were derived from NCBI genome viewer. Bars of red, purple, and black indicate regions of protein coding, transfer/ ribosomal RNA coding, and regulatory D-loop, respectively.

**Figure 2:**
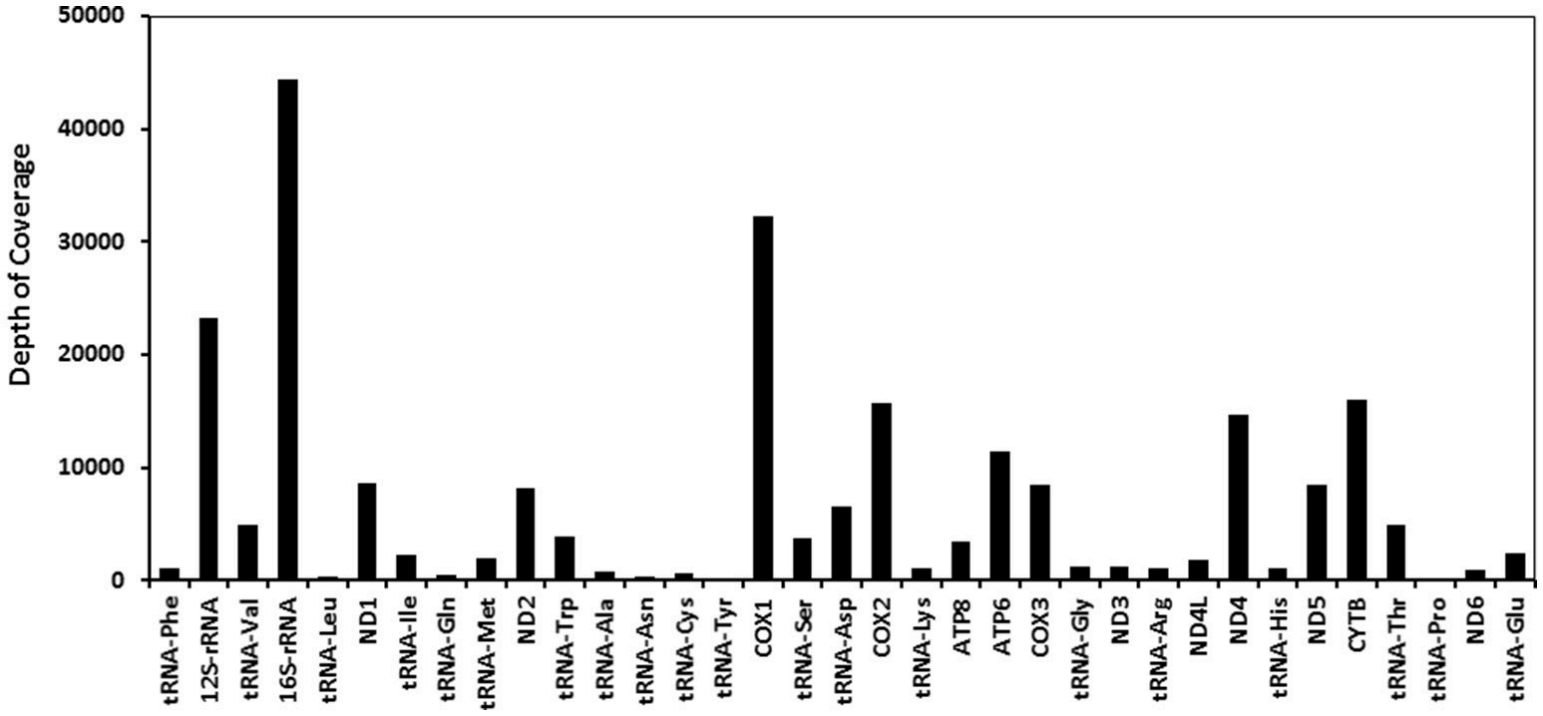
Relative expression of total mitosRNAs in each mitochondrial gene. X axis indicates mitochondrial coding regions, while Y axis represents depth of coverage (raw sequence read counts as results of small RNA sequencing).

Only four mitosRNAs (gga_mitosRNA_L_6542, gga_mitosRNA_L_16177, gga_mitosRNA_L_16707, and gga_mitosRNA_L_16745) were derived from the light strand while 113 mitosRNAs were derived from heavy strand mitochondrial DNA (Supplementary Table 1). Similar to findings by Ma et al. (2016), the mitosRNAs were not evenly distributed across the mitochondrial genome (Figure 1). Within the D-loop, rRNA, tRNA, and protein coding regions, the protein- and rRNA coding regions produced much higher number of mitosRNAs (Figure 2), which differs from reports in other species that showed higher mitosRNA frequencies in tRNA coding regions (Lee et al., 2009; Mercer et al., 2011; Kumar et al., 2014; Goodarzi et al., 2015; Hirose et al., 2015; Ma et al., 2016). The unique condition of higher mitosRNAs in mitochondrial protein- and rRNA genes suggests that mitochondrial functions of electron transport chain associated with energy expenditure and OXPHOS in avian muscle may be regulated by unique mechanisms different from other species. Interestingly, our earlier results of global gene expression, in which same RNA samples were used, indicated that muscle growth and fiber composition may be regulated by distinct mitochondrial functions in chicken that might be different from other species (Kong et al., 2017).

### Differentially expressed (DE) mitosRNA in PeM compared to BPR

Since our previous global gene expression study (Kong et al., 2017) indicated differential functions of mitochondria in muscles between PeM (rapid growth and large muscle mass) and foundational BPR chickens (slow growth and small muscle mass), differentially expressed (DE) mitosRNA between PeM and BPR were analyzed in the present study. Of the 117 potential mitosRNAs, 44 DE mitosRNAs with adjusted *p*-values (FDRs) <0.05 were identified. All of the DE mitosRNAs were upregulated in PeM breast, except those that were encoded by the 16S-rRNA gene region (Table 2). Similar to the abundance of total mitosRNAs, only 3 mitosRNAs (gga_mitosRNA_H_4040, gga_mitosRNA_H_8272, and gga_mitosRNA_H_8277) were derived from tRNA coding region while 41 mitosRNAs were derived from mitochondrial protein- and rRNA coding regions (Table 2). The mitosRNAs may be expected to regulate expression and stability of mRNA that is the origin of the designated mitosRNA (Ro et al., 2013; Ma et al., 2016). To validate small RNAseq results, a subset of 12 mitosRNAs that were the most abundant in each gene were subjected to qPCR (Table 3). Results indicated that expression levels for 11 of 12 mitosRNAs were well-matched between small RNAseq and qPCR analyses in terms of the direction and magnitude of change (Table 3). One possible reason why the DE value of a mitosRNA was not matched between RNAseq and qPCR may be related to the use of different normalization approaches between the two methods. The mitosRNAs showing similar DE predictions can be considered the most important of our reported data.

**Table 2:** The differentially expressed mitosRNAs in PeM (*N* = 6) compared to BPR (*N* = 6).

**Name**	**RNA sequence**	**Length**	**Region**	A^*^	M^**^	**FDR**
gga_mitosRNA_H_1426	GTAGCCCAAGACGCCTTGCTTAAGCC	26	12S rRNA	10.17	1.79	0.0376
gga_mitosRNA_H_1781	ATCACACATGTATCCGCCTGAGAACTACGAGCACA	35	12S rRNA	13.33	1.66	0.0395
gga_mitosRNA_H_1816	AACGCTTAAAACTCTAAGGACTTGGCGGTGCCCCA	35	12S rRNA	9.98	2.23	0.0143
gga_mitosRNA_H_2346_1	CTTGCCCCCCCTCTAGCCCGACAAACTAGTA	31	16S rRNA	10.72	−1.47	0.0165
gga_mitosRNA_H_2346_2	CTTGCCCCCCCTCTAGCCCGACAAACTCATAC	32	16S rRNA	10.11	−1.50	0.0218
gga_mitosRNA_H_2346_3	CTTGCCCCCCCTCTAGCCCGACAAACTCCTAC	32	16S rRNA	12.02	−1.46	0.0451
gga_mitosRNA_H_2346_4	CTTGCCCCCCCTCTAGCCCGACAAACTCGCACC	33	16S rRNA	11.59	−1.44	0.0218
gga_mitosRNA_H_2346_6	CTTGCCCCCCCTCTAGCCCGACAAACTCGTACCCTTAACATAAAAA	46	16S rRNA	17.95	−1.19	0.0387
gga_mitosRNA_H_2346_8	CTTGCCCCCCCTCTAGCCCGACAAACTCGTCCC	33	16S rRNA	12.23	−1.62	0.0285
gga_mitosRNA_H_2346_10	CTTGCCCCCCCTCTAGCCCGACAAACTGGTA	31	16S rRNA	10.49	−1.54	0.0165
gga_mitosRNA_H_2346_11	CTTGCCCCCCCTCTAGCCCGACAAACTTGTA	31	16S rRNA	11.21	−1.91	0.0156
gga_mitosRNA_H_3067	TTATTAACAGAACTCAACTTATACCCCCA	29	16S rRNA	11.48	2.36	0.0080
gga_mitosRNA_H_3421	ATAAGACGAGAAGACCCTGTGGAACTTTAA	30	16S rRNA	11.55	1.75	0.0218
gga_mitosRNA_H_4040	TACCCCGGACATGACCCTGCCCACCCTAACA	31	tRNA-Leu	13.55	1.16	0.0156
gga_mitosRNA_H_4554	TTAAGCACCCTGGCCATCACCCAAGAACCC	30	ND1	11.53	3.91	0.0156
gga_mitosRNA_H_4957	TAACCCTAGCCTTATGCCTCTGACATACCAGCATACC	37	ND1	11.59	2.39	0.0092
gga_mitosRNA_H_5727	CTAATCGGAGGCTGAATGGGCCTAAACC	28	ND2	11.33	2.20	0.0218
gga_mitosRNA_H_5954	AATACTAAATGCAACTGTAATACTAACCC	29	ND2	11.27	2.82	0.0048
gga_mitosRNA_H_6276_1	CTACAGAAACTTAGGATTAACTGTCACC	28	ND2	13.71	1.62	0.0156
gga_mitosRNA_H_6678	AACCACAAAGACATTGGCACTCTTTACCT	29	COX1	10.37	2.84	0.0048
gga_mitosRNA_H_7156	TAAAACCCCCCGCACTGTCACAATACC	27	COX1	11.30	1.63	0.0048
gga_mitosRNA_H_8083	GAAAAGTACTCCAACCCGAATTAACT	26	COX1	11.86	1.76	0.0156
gga_mitosRNA_H_8272	AAACCAATTACATAGACCTGTCAAGACTA	29	tRNA-Asp	12.71	1.66	0.0349
gga_mitosRNA_H_8277	AATTACATAGACCTGTCAAGACTAA	25	tRNA-Asp	11.47	1.76	0.0080
gga_mitosRNA_H_8375	CATCATAGAAGAGCTCGTTGAATTCCAC	28	COX2	10.30	2.60	0.0218
gga_mitosRNA_H_8487	AACACCGTAGATGCCCAAGAAGTTGAACTAATCTGAACC	39	COX2	12.41	2.25	0.0221
gga_mitosRNA_H_9163	AAACTTCTTTCATTCACTCTAACAAACAACCCTGCA	36	ATP8	12.92	1.65	0.0080
gga_mitosRNA_H_9235	TGACCATGAACCTAAGCTTCTTCGACC	27	ATP8	8.31	2.04	0.0354
gga_mitosRNA_H_9601	AACCCTCCGCCTCCTTAGGACACCTACTCCCTGAAGGCACCCC	43	ATP6	12.18	2.04	0.0266
gga_mitosRNA_H_9757	AACTTATCTCTACAGCCACAATCGCCCTACTACC	34	ATP6	13.36	1.44	0.0080
gga_mitosRNA_H_9800	ATCAATCTCCGCCCTAACGGCACTCA	26	ATP6	10.67	2.89	0.0080
gga_mitosRNA_H_9871	AAGCCTACGTCTTCGTCCTCCTCCTAAGCCTCTACTTACA	40	ATP6	12.85	1.50	0.0156
gga_mitosRNA_H_10617	ACTTCGGATTTGAAGCAGCAGCCTGATACTG	31	COX3	10.57	2.68	0.0218
gga_mitosRNA_H_11350	AAACCCCATCATTCGCCCTTGTACC	25	ND4_L	9.38	2.03	0.0218
gga_mitosRNA_H_11633	AAAACCCTAACCCTCTGAACAGGCATAGACCA	32	ND4	11.42	2.36	0.0049
gga_mitosRNA_H_11664	AAATCTCAACTCCCCTCTTAGTACTCTCCTGC	32	ND4	12.93	2.05	0.0049
gga_mitosRNA_H_11665	AATCTCCACTCCCCTCTTAGTACTCTCCTGC	31	ND4	10.70	2.65	0.0005
gga_mitosRNA_H_11905	AGAACGACTTAGCGCAGGCATTTACC	26	ND4	9.48	1.55	0.0218
gga_mitosRNA_H_12707	CTCTACATACTACTCTCAACCCAACGAGGCACTC	34	ND4	10.09	1.56	0.0218
gga_mitosRNA_H_12766	CAACTCAAACACTCGAGAACATCTTCTCATAACCC	35	ND4	10.11	2.41	0.0218
gga_mitosRNA_H_12824	CTCATCCTCAAACCAGAACTAATCTCAGGAACCC	34	ND4	11.78	2.58	0.0080
gga_mitosRNA_H_15821	AATCTAAACAACGAACAATAACCTTCCGACC	31	CYTB	12.75	1.98	0.0048
gga_mitosRNA_H_15860	AAACCCTATTCTGACTTCTAGTAGCCAACC	30	CYTB	11.93	1.87	0.0218
gga_mitosRNA_H_15904	TGAATCGGAAGCCAACCAGTAGAACACCCC	30	CYTB	9.53	3.17	0.0218

* *A denotes Log2 value of average expressions*.

** *M means Log2 fold change of differential expressions*.

**Table 3:** Comparison of fold changes (FC) between RNAseq and qPCR.

**Gene symbol**	**PeM (*****N*** = **6) vs. BPR(*****N*** = **6)**				
	**RNAseq**		**qPCR**		**Gene**
	FC^*^	**FDR**	FC^*^	***p*-value**	
gga_mitosRNA_H_1781	3.17	0.0395	5.67	0.0007850	12 S rRNA
gga_mitosRNA_H_2346_6	−2.28	0.0387	−1.36	0.0476534	16 S rRNA
gga_mitosRNA_H_4040	2.23	0.0156	2.02	0.0007420	tRNA-Leu
gga_mitosRNA_H_4957	5.23	0.0092	3.55	0.0000156	ND1
gga_mitosRNA_H_8083	3.40	0.0156	3.24	0.0014579	COX1
gga_mitosRNA_H_8272	3.17	0.0349	−2.40	0.0255489	tRNA-Asp
gga_mitosRNA_H_8487	4.77	0.0221	4.22	0.0001418	COX2
gga_mitosRNA_H_9163	3.14	0.0080	3.03	0.0000015	ATP8
gga_mitosRNA_H_10617	6.41	0.0218	2.48	0.0007107	COX3
gga_mitosRNA_H_11350	4.09	0.0218	4.39	0.0000091	ND4_L
gga_mitosRNA_H_11664	4.13	0.0049	4.62	0.0000016	ND4
gga_mitosRNA_H_15821	3.94	0.0048	5.81	0.0000010	CYTB

* *Values denote linear fold changes*.

### Comparison between DE MitosRNA and DE mRNA

In order to determine the abundance of total mitosRNAs encoded by each mitochondrial gene, the read counts of all mitosRNAs in a gene region were added to represent a gene and then compared between PeM and BPR. In these results, we noted the same differential pattern as that shown in the DE individual mitosRNA (data not shown). Since DE mitosRNAs were mostly found in protein coding genes (mRNA), the DE mitosRNAs were compared to gene expression derived from the RNAseq analysis conducted on the same muscle samples (Kong et al., 2017). In the RNAseq analysis conducted in the previous study (Kong et al., 2017), differential expression of mitochondrial mRNA was not observed between PeM and BPR (Table 4). However, when data from both DE mitosRNAs and non-DE mitochondrial mRNA were considered, distinct phenotypes (rapid growth, greater muscle mass, fiber composition change) associated with mitochondrial functions shown in modern PeM may be regulated by higher mitosRNA production than the mitochondrial mRNAs (Table 4). Thus, potential regulatory activities of mitosRNAs may influence the stability of the source mRNA and, in turn, ultimately affect the protein abundance and protein functionalities in electron transport chain for OXPHOS and energy expenditure. Future studies on mitosRNAs in chicken muscles will be important to characterize the regulation of differential mitochondrial protein abundance and their functions influenced by mitosRNAs.

**Table 4:** Comparison of differential expression of mitosRNA and mRNA in gene regions of mitochondrial genome in PeM breast muscle (*N* = 6) compared to BPR (*N* = 6).

**Gene Region**	**mitosRNA**		**mRNA**	
	**Log_2_FC**	***p*-value**	**Log_2_FC**	***p*-value**
ATP6	1.33	0.00013	0.26	0.195682
CYTB	1.54	0.0003	0.31	0.129304
ND2	1.51	0.00032	0.36	0.198144
ND4	1.30	0.00054	0.05	0.753202
ND1	1.34	0.00116	−0.02	0.928934
ATP8	1.09	0.00134	0.58	0.078447
ND5	0.94	0.00442	0.14	0.306487
COX1	1.09	0.00481	0.09	0.68997
COX3	0.83	0.00697	−0.07	0.800796
ND4L	1.09	0.00889	0.01	0.978282
COX2	1.02	0.01297	na	na
ND3	0.90	0.05364	0.17	0.552786
ND6	−0.11	0.5783	0.83	0.003621

### Differential abundance of mitosRNAs correlated with mitochondrial contents

**Figure 3:**
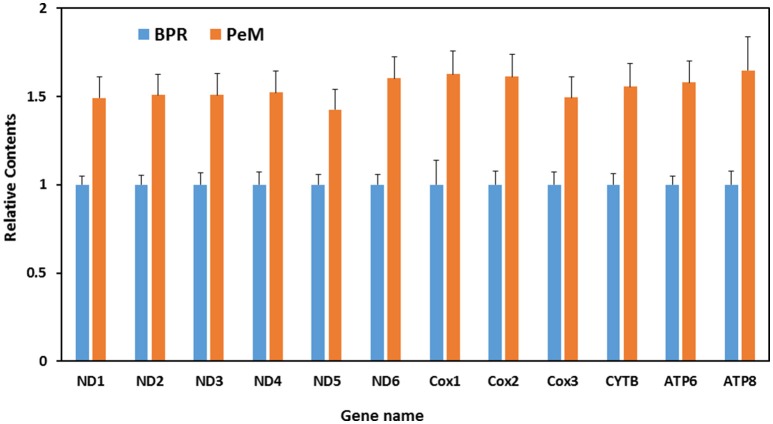
Differential mitochondrial contents. X axis indicates mitochondrial protein coding regions, while Y axis represents ratio of DNA contents calculated by DNA contents of PeM divided by DNA contents of BPR. Data are the mean ± S.E. from six individual samples per group.

While higher abundance of mitosRNAs in PeM could be a function or result from higher mitochondrial content in the muscle tissue, a global transcriptomic study conducted on the same tissue samples reported a significant skew of the mitochondrial proteome in the BPR muscle compared to the PeM which suggests that the mitochondrial content was greater in the BPR tissue than in the PeM tissue (Kong et al., 2017). This finding conflicts with the results of the present study. For this reason, mitochondrial contents were determined by qPCR with muscle DNA and primers for various gene regions (e.g., ND1, ND2, ND3, ATP6, ATP8, COX2, COX3 etc.) of mitochondrial genome, compared with stationary contents of nuclear DNA sequences (GAPDH). When the same amount of DNA was used, the average contents of nuclear genes were very similar among samples in a group and even between groups of PeM and BPR (data not shown), suggesting nuclear DNA copy numbers are the same between samples. In contrast, the average content of mitochondrial genes are slightly higher in PeM compared to BPR (Figure 3). Mitochondrial DNA sequences were ~1.58-fold higher in average throughout the mitochondrial genes in PeM samples compared to BPR muscles, indicating a modest increase of mitochondrial content in modern breeds selected for growth and FE (Figure 3). This result supports the argument that the higher abundance of mitosRNAs in PeM muscles may be derived from the higher mitochondrial contents. Further studies are warranted to fully characterize functions of the lower abundance of mitosRNAs derived from 16S ribosomal RNA in PeM.

According to an earlier transcriptomic investigation with the same tissue samples (Kong et al., 2017), upregulated muscle-related genes suggested a possible higher slow muscle fiber composition in PeM compared to BPR. In addition, an independent report by Mutryn et al. (2015) suggested that wooden breast myopathy (which in commercial broilers showed a dramatic upregulation of troponin I1, myoglobin, myosin binding protein C1, and myozenin 2 genes compared to unaffected muscle) may undergo fast-to-slow fiber type switching. Slow-twitch or oxidative fibers usually possess a higher mitochondrial content, slower contraction rates, increased reliance on OXPHOS, higher fatigue resistance, and higher representation of postural muscles. In contrast, fast-twitch or glycolytic fibers have lower mitochondrial content, rapid contractions, decreased reliance on OXPHOS, low resistance to fatigue, and high representation in muscle groups used for directional movement (Mishra et al., 2015). Chickens have white breast muscle composed primarily of fast twitch glycolytic fibers bearing low mitochondrial content (e.g., Kiessling, 1977). Thus, the fact that higher mitosRNAs with higher mitochondrial contents retained in PeM breast may be indicative evidence for fiber type switching to increase slow-twitch fibers in modern broiler breasts.

In mammals, mitosRNAs are known to be generated by unidentified ribonucleases retained in mitochondria since mammalian mitosRNAs contain 5′ phosphate and 3′ hydroxyl termini that are found in microRNA and small nucleolar RNAs (snoRNAs) (Ro et al., 2013). Depending on the orientation, sense mitosRNAs enhance the expression of host mitochondrial genes by targeting anti-sense transcripts. In contrast, anti-sense mitosRNAs inhibit mitochondrial gene expression by targeting sense transcripts (Ro et al., 2013). Chicken muscle mitosRNAs, which were significantly expressed in this study, are all sense orientations: suggesting that the mitosRNAs may enhance the expression of mitochondrial genes by suppressing antisense RNAs in modern PeM broilers compared to unselected BPR. Although the mRNA expression of mitochondrial genes were not different between PeM and BPR, mRNA stability may be influenced by mitosRNA and, in turn protein production may be affected. Thus, further studies on mitochondrial protein encoding and synthetic machinery may need to be conducted in the future in order to fully understand the role that mitosRNA plays in growth and development of muscle in modern broilers.

## Author contributions

WB and BCK conceived and designed this study. BK, SS, DS, and SK analyzed small RNAseq data and performed qPCR. BM, SO, CO, and NA conduct the animal care, sample collection, and data analyses. JP and JK contribute to data analyses associated to mitochondria. The paper was written through contributions and critical review of the manuscript by all authors (WB, BK, SS, DS, BM, SO, JP, SK, CO, NA, JK, and BCK).

### Conflict of interest statement

The authors declare that the research was conducted in the absence of any commercial or financial relationships that could be construed as a potential conflict of interest. The reviewer DC and handling Editor declared their shared affiliation.

## Supplementary material

Supplementary Table 1 The 117 chicken muscle mitosRNA.
